# Assessment of Performance of the Industrial Process of Bulk Vacuum Packaging of Raw Meat with Nondestructive Optical Oxygen Sensing Systems

**DOI:** 10.3390/s18051395

**Published:** 2018-05-02

**Authors:** Caroline A. Kelly, Malco Cruz-Romero, Joseph P. Kerry, Dmitri P. Papkovsky

**Affiliations:** 1 School of Biochemistry and Cell Biology, University College Cork, Cavanagh Pharmacy Building, College Road, Cork T12 K8AF, Ireland; carolineakelly@gmail.com; 2 Food Packaging Group, School of Food and Nutritional Sciences, University College Cork, Food Science Building, College Road, Cork T12 K8AF, Ireland; m.cruz@ucc.ie (M.C.-R.); joe.kerry@ucc.ie (J.P.K.)

**Keywords:** packaged raw meat, bulk vacuum packaging, nondestructive assessment, residual oxygen, phosphorescent oxygen sensor, QC/QA, food safety and process optimisation

## Abstract

The commercially-available optical oxygen-sensing system Optech-O_2_ Platinum was applied to nondestructively assess the in situ performance of bulk, vacuum-packaged raw beef in three ~300 kg containers. Twenty sensors were attached to the inner surface of the standard bin-contained laminate bag (10 on the front and back sides), such that after filling with meat and sealing under vacuum, the sensors were accessible for optical interrogation with the external reader device. After filling and sealing each bag, the sensors were measured repetitively and nondestructively over a 15-day storage period at 1 °C, thus tracking residual oxygen distribution in the bag and changes during storage. The sensors revealed a number of unidentified meat quality and processing issues, and helped to improve the packaging process by pouring flakes of dry ice into the bag. Sensor utility in mapping the distribution of residual O_2_ in sealed bulk containers and optimising and improving the packaging process, including handling and storage of bulk vacuum-packaged meat bins, was evident.

## 1. Introduction

Fresh raw meat represents a large and important segment of fast-moving packaged food products produced commercially on a large scale, for many different types of meats, portion sizes, and formats. To ensure the delivery of high quality and safe products to market, prevent meat deterioration, and extend product shelf life, the employment of modified atmosphere packaging, including vacuum packaging, is undertaken. By tailoring gas composition within the package, packaging materials used, and processes employed, microbial growth and other degradative processes can be inhibited and/or product appearance improved [1,2]. For example, some red meats such as steaks require medium to high levels of O_2_ to allow oxygenation of the endogenous myoglobin which maintains the aesthetically pleasing red colour. In contrast, lipid (and other) oxidation-led reactions necessitate low O_2_ levels to prevent a decline in product quality. Residual O_2_ is a useful indicator of package integrity, packaging material defects, or signs of physical abuse of packaged goods during processing, storage, and transportation.

Close monitoring of O_2_ levels within packages enables the detection of the issues described, highlights steps that need to be addressed in order to bring packaging and related processes under control, and directs attention to defective packs where the required protective atmosphere has been lost [3]. Such products can be repackaged before they leave the factory or be removed from the line, thereby preventing any low-quality and potentially unsafe goods from reaching the retailer and consumer and circumvent other issues such as customer complaints, product removal from supermarket shelves, logistical issues such as product retrieval by manufacturers from the market, and so on.

Currently used packaging approaches do not allow for nondestructive, real-time, repetitive measurement of the environmental conditions within individual packs after they have been produced. Usually, a certain proportion of packs are sampled statistically and assessed for O_2_ content by destructive methods such as gas chromatography or needle-type gas analyzers which can measure O_2_ and CO_2_ [2]. These methods are invasive; incapable of detecting all faulty or insufficient-quality packs, which have been shown to be rather frequent [4,5]; often require a skilled operator; consume the analyte measured; poorly suited for vacuum-packaged samples (shrunk and possessing no headspace); in addition to rendering the pack unsuitable for return to the line or release to the market.

Phosphorescent O_2_ sensors, such as those developed by Mocon, Presens, Oxysense, and other vendors [3], can help address the issues highlighted above. These systems can provide simple, accurate, nondestructive, real-time measurement of residual O_2_ levels in multiples of sealed containers for a wide range of packaged food and beverage products. The advantages of using these types of sensor is that they can be used on a disposable or continuous basis and offer major advantages over alternative systems in the analysis of residual O_2_ levels. The sensors provide quick, quantitative, contactless readout of O_2_ concentration and can serve a multitude of analytical and quality control/quality assurance (QC/QA) tasks [6,7]. Such sensors currently cost a few dollars each and efforts are being made to make them even more affordable, calibration-free, and capable of being produced on a large-scale basis and incorporated ultimately in every pack [8,9]. A number of studies employing such O_2_ sensors have been conducted using food products, such as ham [10], beef [5], cheese [11], lettuce [12], bread [13], convenience foods [14], and for troubleshooting within cheese production processes [15] and to measure the depletion of O_2_ in raw beef and chicken packs [16]. However, no studies of which we are aware have been performed using such sensor systems to assess the large-scale, bulk packaging of beef in a commercial meat processing plant. On the other hand, in bulk vacuum packaging of raw meat, significant heterogeneity of residual O_2_ levels in packaged samples is anticipated, which may be caused by slow filling of the container with the product, trapping and creating pockets of air between meat pieces and layers of meat, incomplete flushing of large product-filled containers with inert gas, and subsequent sealing under vacuum.

In this study, we set out to demonstrate the effective use of the commercial Optech-O_2_ Platinum sensor system to control the quality of bulk packaging of raw beef in an Irish commercial beef processing plant.

## 2. Materials and Methods

### 2.1. Preparation of Containers with Sensors and Meat

**Figure 1:**
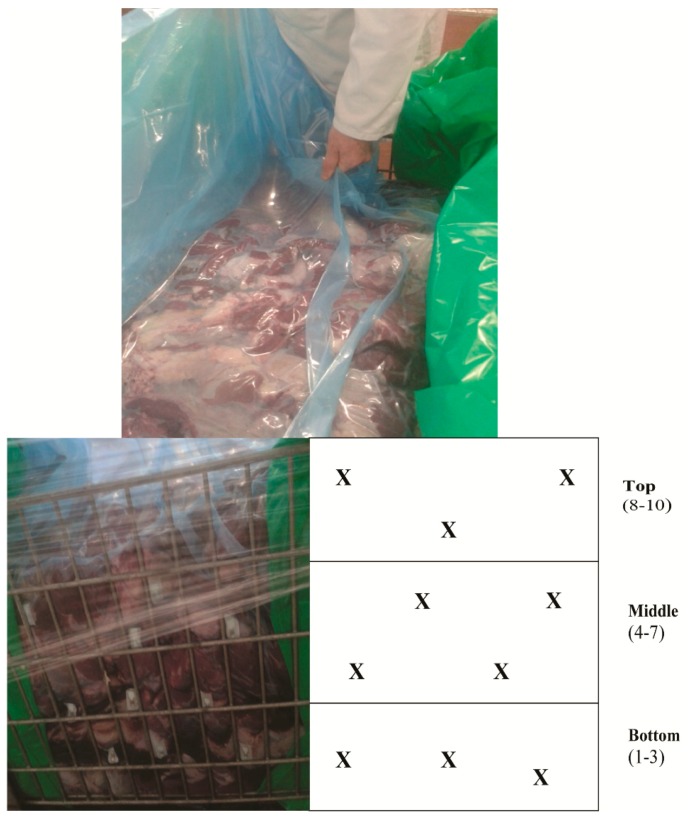
Top view of a bulk bin with vacuum-packaged meat (**top**) and its side view in a bin (**bottom left**) showing the location of individual O_2_ sensors (**bottom right**).

Twenty phosphorescent O_2_ sensor stickers (Mocon, Minneapolis, MN, USA) were attached to the inner side of a bulk, laminate plastic-based pouch which was designed for vacuum packing and employment within a steel frame storage bin for the bulk transportation of beef carcass off-cuts. The sensors were distributed evenly inside the laminate pouch: ten sensors were facing the front side and ten were facing the back side of the bin (these sides contained metal grids, and the other two sides were covered with metal sheets). The sensors were positioned such that after bulk pouch filling with meat and sealing under vacuum, they could be accessed for reading purposes through the use of an external handheld Optech-O_2_ Platinum reader (Mocon, Minneapolis, MN, USA). This container was then filled with approximately 300 kg of forequarter meat. Flakes of dry ice were applied periodically between layers of meat. After filling, the container was left to rest for 45 min to allow the evaporating dry ice to displace residual O_2_ from within the pouch. Then, the whole container was moved by forklift to a vacuum-packaging unit, vacuum packed, and sealed. Images of a bulk bin container containing meat and located sensors are shown in Figure 1. Thus, each sensor reported on residual O_2_ levels in the specific corresponding area of the package within which it was situated. A total of three bins were produced and analysed in this manner.

### 2.2. Storage and Measurement of O_2_ Levels

After the packaging process was completed, the bulk bin container was moved to a chilled warehouse where it was stored at 1 °C for 15 days. Prior to the measurement of the O_2_ sensors in bins, the Optech-O2 Platinum system was calibrated at 1 °C with a dedicated calibration card (CalCard^®^, Mocon). On Days 1, 3, 7, 11, and 15, the bins were brought to the processing hall, where temperature was ~12 °C, and O_2_ readings from each sensor were quickly taken with the Optech^®^ reader by bringing it close to the sensor (<10 mm distance) and initiating the reading. The instrument measured the phosphorescence signals (intensity and lifetime) as well as sample temperature with a built-in infrared (IR) probe, performed the necessary correction and calculations, displayed O_2_ concentration (in % or kPa units) in the corresponding region of bag, and stored the data. Measurement of each sensor took between 1 and 3 s to complete.

## 3. Results and Discussion

### Design of the Sensor Trial with Bulk Vacuum-Packaged Meat

In food packs that contain headspace, such as modified atmosphere packaged (MAP) foods, it is usually sufficient to employ one sensor to gather information about the average residual oxygen levels present within the whole pack. However, in products such as vacuum-packaged meats, readings from one sensor at a specific location may not be representative, since the sensor is only capable of detecting the presence of oxygen in the immediate locality to where the sensor is positioned, and consequently, the ability to determine O_2_ concentration and gas exchange within such a pack is restricted, since a headspace is absent [17]. Even for small vacuum packs, this creates a problem, since physical damage or faults affecting remote areas of the package are not always detectable with one sensor, since its response is mediated by O_2_ diffusion.

For large packages, such as bulk vacuum-packaged systems, one can also anticipate significant heterogeneity in residual O_2_ levels throughout the package. This can arise from the slow filling of the container with the product, trapping air and creating air pockets between individual products and product layers, difficulties with efficient flushing of the large product-filled container with inert gas, and its subsequent sealing under vacuum. All of these issues apply to the bulk vacuum packaging of primal meat cuts destined for subsequent processing. From a production perspective, it is critically important to be able to visualise and control such heterogeneities in order to maintain product safety and quality. This, in turn, can open the way to optimising and minimising these heterogeneities, and by so doing, control the residual oxygen level for the whole pack.

One simple way to address these challenges is to incorporate multiple O_2_ sensors per pack which can report nondestructively on local O_2_ levels in various regions of the pack. Periodic measurement of these sensors would allow one to visualise maps of O_2_ distribution in such packages and understand their dynamics during product handling and shelf life. With oxygen sensors becoming more affordable, accurate, and calibration-free, such trials, even with very large bulk product packs employing numerous sensors, are now becoming more simple to use, informative, and commercially justifiable. From a sustainability and environmental perspective, this means that the products examined are not wasted and the sensors can also be recovered, cleaned, and reused, if required.

Thus, we introduced an array of individual O_2_ self-adhesive-based sensors in the large, laminate plastic-based pouches employed for bulk vacuum packaging of fresh meat, as described previously, to assess the efficiency of the packaging procedure, optimise it, and ensure that residual O_2_ content, quality, and safety of the packaged meat were all within acceptable limits.

A total of three bins, each filled with approximately 300 kg of fresh beef, were produced and analysed in this manner. The top and side views of a bulk bin containing vacuum-packaged meat and sensors (a total of 10 sensors located on the front and back side of the vacuum-packaged laminate pouch) are shown in Figure 1.

In the first experimental trial (Bin 1), sensors detected that the laminate pouch was not vacuum-packaged correctly owing to an insufficient vacuum being pulled, thereby allowing some of the CO_2_ atmosphere generated by employment of dry ice to be replaced with air (high O_2_). This necessitated the package being opened again and resealed under vacuum. Owing to this containment fault, residual O_2_ levels in Bin 1 were relatively high.

Individual O_2_ sensor readings from Bin 1 are shown in Table 1. On the day of filling (Day 1), three out of 20 sensors were masked by the metal grid of the container and were unreadable. However, on subsequent Days 3, 8, 11, and 15, they became accessible for measurement owing to slight movements of the bag inside the bin following bin transportation within the factory by forklift. On Day 1, a significant number of sensors (9 out of 17) showed residual O_2_ levels of >1%, with two sensors indicating the presence of >6% O_2_. Mean O_2_ levels on Day 1 were determined to be 1.8 ± 2.3% (mean and SD values). Such levels were deemed unacceptably high, as typical specifications for small vacuum packs of beef are <0.5% O_2_ [5].

It is worth noting that during the O_2_ measurements in the bins, sample/sensor temperatures typically ranged from 3.0 to 3.8 °C, being close to Optech’s normal operational range (5–40 °C, according to vendor specifications). In addition, with the calibration at 1 °C using the Mocon CalCard, the Optech system provided reliable and accurate O_2_ readings under experimental conditions used in this study. Thus, individual sensors measured under identical experimental conditions (1 °C, 20.86% O_2_) showed O_2_ values 20.6 ± 0.8% (mean and SD). They are shown in Supplementary Materials, Figure S1.

On Day 3, O_2_ values decreased significantly (0.7 ± 1.1), as determined by the majority of sensors; however, five out of 20 sensors still detected >1% O_2_ (Table 1). From Day 8 onwards, the majority of sensors provided O_2_ readings below 0.3%. Mean O_2_ values on the subsequent days stabilised and remained at around 0.3–0.4% (Table 1).

The industrial packaging process is aimed at bringing the environment of the pack from the initial 20.86% of O_2_ (ambient air) down to zero levels (or as close as possible). This is particularly challenging for large and heterogeneous samples and multistep packaging processes, such as those used in our meat bins. Residual microbubbles of air trapped between meat pieces and incomplete flushing or displacement of air with CO_2_, if they occur, are expected to have significant impact on local residual O_2_ levels.

Our experimental results with O_2_ sensor arrays in Bin 1 satisfactorily prove this and reveal prominent sample heterogeneity. They also prove that single-point O_2_ measurements, currently used in process control, are not efficient and less representative for such samples. Under identical experimental conditions (1 °C, 20.86% O_2_), individual sensors show variability of ±0.05% O_2_ (see Mocon product specifications and Supplementary Materials, Figure S1), therefore sensor variability has a very minor contribution to the variability or sensor readings in the bins.

The technical and packaging processing issues highlighted in the experimental Bin 1 trial were partly addressed when conducting the second experimental trial employing Bin 2. However, vacuum packaging and sealing were problematic again, and Bin 2 had to be opened and resealed as described previously for Bin 1. As a consequence, Bin 2 indicated lower O_2_ levels than Bin 1 (mean values were less than 1.2% throughout the study), but still above the anticipated, required, and achievable range (Table 2). Again, eight sensors out of 20 showed O_2_ levels exceeding 1%.

**Figure 2:**
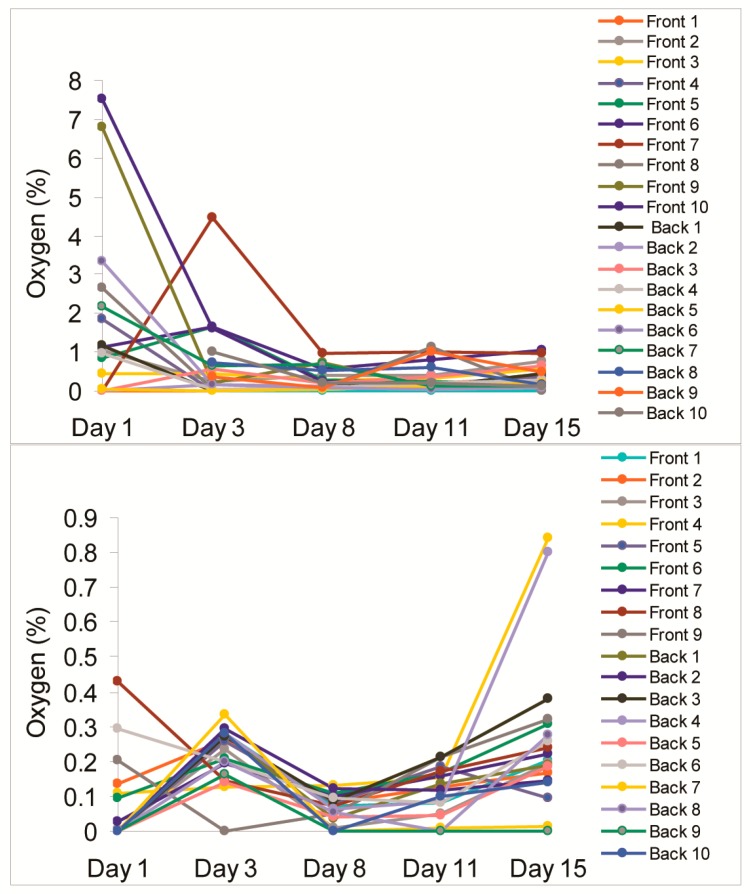
Mean O_2_ readings obtained from sensor stickers placed in Bins 1 (**top**) and 3 (**bottom**) over a 15-day storage period.

In the final experimental trial involving Bin 3, in which air was properly displaced with CO_2_ (dry ice) and sealed correctly following efficient vacuum application, the lowest O_2_ levels were determined of the three experimental bins (Table 3). Mean residual O_2_ levels within the bulk vacuum pack stayed in the range of 0.07–0.22% over the initial 11 days of storage. In addition, on Day 15 of storage, two sensors showed marked increase in O_2_ (Figure 2), resulting in significantly elevated mean values and SD on that day. We credited the latter effects to the potential microrupture of the pouch while being transported from the warehouse to the production floor by forklift or following the release of air from gas pockets trapped within the bulk meat mass somewhere close to these sensors. One additional sensor in Bin 3 was rendered unreadable by puckering of some of the plastic packaging through meat movement within the container.

Having measured residual O_2_ values in all three bins from all usable sensors, nondestructively, and repetitively over three repeated 15-day storage periods, we further analysed these results by comparing all three data sets. Figure 2 shows the dynamics of O_2_ readings taken from all individual sensors located in Bin 1 (top) and Bin 3 (bottom). One can see that the O_2_ scale for the packaging process carried out for Bin 1 is greater by almost one order of magnitude when compared to Bin 3, indicating the difference between ineffective packaging application and effective packaging application.

The majority of O_2_ readings from the sensors in Bins 1 and 2 show a consistent downward trend over time (see individual and mean O_2_ values in Table 1, Table 2 and Table 3). We attribute this to oxygen consumption by the meat and gradual equilibration of sample macroenvironment. Occasional jumps in O_2_ readings are likely due to the handling of the bin with a forklift between the measurements. This can dislocate pieces of meat and sensors within the bin and/or release some air trapped in areas adjacent to the sensor, and thus affect the O_2_ readings.

**Figure 3:**
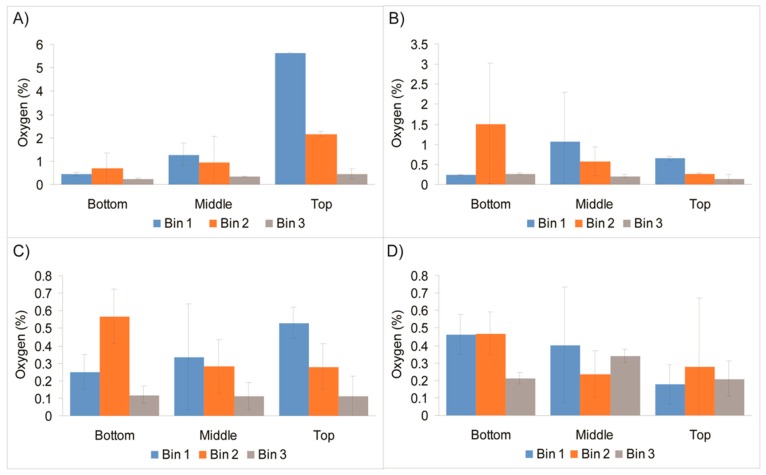
Comparison of mean O_2_ readings in top, middle, and bottom parts of Bins 1, 2, and 3, measured on Day 1 (**A**), Day 3 (**B**), Day 11 (**C**), and Day 15 (**D**).

Figure 3 shows average O_2_ readings and SD for sensors located in the three primary location areas (bottom, middle, and top) within each bulk vacuum-packaged pouch, which reveal trends in O_2_ distribution within each bin. In Bin 1, on Day 1, O_2_ concentration increased from the bottom to the top of the bulk pouch. The top section of the bin is seen as most vulnerable to O_2_ ingress, air displacement with CO_2_, and the quality of vacuum sealing applied. In particular, Bins 1 and 2 showed the highest O_2_ levels in the top section of the bins, most likely due to ineffective packaging and the requirement to revacuum package and reseal. However, by Days 3, 8, and 11, the random distribution of O_2_ concentrations throughout Bins 1 and 2 were similar. Day 15 showed a shift in O_2_ distribution from previous storage days, possibly induced by the movement of the bulk-containing bins from the warehouse to the production floor. Bin 3 possessed a more uniform O_2_ distribution pattern from the top of the pack to its bottom throughout the 15-day storage period.

## 4. Conclusions

Incorporating an array of individually addressable phosphorescence-based O_2_ sensor stickers in large bins of raw meat (300 kg each) which undergo bulk packaging under vacuum has enabled nondestructive, contactless monitoring of residual O_2_ levels in the various parts of the bin throughout a 15-day storage period. Such measurements performed with commercial O_2_ sensors and handheld detection instrumentation helped to map O_2_ distribution and dynamics in such commercial bulk meat packs, and to reveal a number of technical issues in the current packaging process. These issues were subsequently addressed and led to a significant improvement in commercial packaging processes, producing much lower and more consistent residual O_2_ levels in bulk meat bins. This analytical approach and optimisation of the packaging process have been adopted by the industrial partner for further operation. The adoption demonstrates how optical O_2_ sensors are effective in detecting process failures and optimising the packaging process, particularly for bulk packaging of raw meat.

## Figures and Tables

**Table 1:** Residual O_2_ level readings (%) for each sensor contained in Bin 1 over a 15-day storage period.

Location	Day 1	Day 3	Day 8	Day 11	Day 15
Front 1	0.00	0.00	0.11	0.22	0.32
Front 2	1.05	0.25	0.42	0.42	0.75
Front 3	0.45	0.46	0.29	0.33	0.57
Front 4	1.85	0.01	0.13	0.14	0.36
Front 5	0.83	1.63	0.27	0.23	0.16
Front 6	1.13	1.64	0.56	0.81	1.06
Front 7	0.00	4.47	0.97	1.02	0.98
Front 8	2.64	0.03	0.08	1.13	0.00
Front 9	6.80	0.21	0.72	0.12	0.08
Front 10	7.53	1.63	0.25	0.15	0.23
Back 1	1.17	0.00	0.11	0.14	0.43
Back 2	0.00	0.17	0.13	0.07	0.10
Back 3	0.00	0.56	0.19	0.34	0.63
Back 4	0.98	0.05	0.08	0.21	0.29
Back 5	0.06	0.02	0.04	0.14	0.19
Back 6	3.35	0.18	0.07	0.04	0.07
Back 7	2.17	0.65	0.70	0.10	0.12
Back 8	N/A	0.72	0.53	0.59	0.17
Back 9	N/A	0.35	0.07	0.99	0.49
Back 10	N/A	1.02	0.21	0.20	0.11
**Mean, % O_2_**	**1.76**	**0.70**	**0.30**	**0.37**	**0.35**
**SD, % O_2_**	**2.26**	**1.05**	**0.27**	**0.35**	**0.31**

N/A: sensor not accessible for measurement.

**Table 2:** Residual O_2_ level readings (%) for each sensor contained in Bin 2 over a 15-day storage period.

Location	Day 1	Day 3	Day 8	Day 11	Day 15
Front 1	0.33	0.50	0.00	0.47	0.36
Front 2	0.00	0.41	0.11	0.76	0.79
Front 3	0.45	0.46	0.11	0.15	0.00
Front 4	0.00	0.36	0.14	0.28	0.00
Front 5	0.00	0.27	0.08	0.31	0.00
Front 6	0.23	0.31	0.59	0.00	0.23
Front 7	0.51	0.36	0.13	0.11	0.34
Front 8	3.52	0.42	0.08	0.82	0.32
Front 9	0.63	0.41	0.17	0.30	0.69
Front 10	2.61	0.00	0.17	0.00	0.66
Back 1	1.24	0.38	0.18	0.39	0.43
Back 2	2.23	7.19	3.36	1.65	0.50
Back 3	0.00	0.17	0.00	0.00	0.74
Back 4	0.66	0.49	0.00	0.55	0.43
Back 5	3.32	0.80	0.26	0.50	0.21
Back 6	0.73	1.71	0.08	0.19	0.43
Back 7	2.19	0.30	0.28	0.32	0.25
Back 8	0.00	0.28	0.14	0.19	0.00
Back 9	3.17	N/A	N/A	N/A	N/A
Back 10	3.07	N/A	N/A	N/A	N/A
**Mean, % O_2_**	**1.24**	**0.82**	**0.33**	**0.39**	**0.35**
**SD, % O_2_**	**1.30**	**1.63**	**0.77**	**0.40**	**0.26**

**Table 3:** Residual O_2_ level readings (%) for each sensor in Bin 3 over a 15-day storage period.

Location	Day 1	Day 3	Day 8	Day 11	Day 15
Front 1	0.00	0.28	0.07	0.08	0.20
Front 2	0.14	0.26	0.08	0.13	0.17
Front 3	0.01	0.24	0.01	0.05	0.20
Front 4	0.11	0.13	0.13	0.16	0.84
Front 5	0.00	0.26	0.08	0.19	0.10
Front 6	0.10	0.21	0.11	0.17	0.31
Front 7	0.03	0.19	0.10	0.16	0.22
Front 8	0.43	0.15	0.07	0.17	0.24
Front 9	0.20	0.00	0.05	0.21	0.32
Back 1	0.00	0.27	0.04	0.13	0.19
Back 2	0.00	0.30	0.12	0.12	0.14
Back 3	0.00	0.27	0.09	0.21	0.38
Back 4	0.00	0.28	0.06	0.09	0.80
Back 5	0.00	0.14	0.04	0.04	0.19
Back 6	0.30	0.20	0.10	0.08	0.26
Back 7	0.00	0.34	0.00	0.01	0.02
Back 8	0.00	0.20	0.10	0.00	0.27
Back 9	0.00	0.16	0.00	0.00	0.00
Back 10	0.00	0.28	0.00	0.10	0.14
**Mean, % O_2_**	**0.07**	**0.22**	**0.07**	**0.11**	**0.26**
**SD, % O_2_**	**0.12**	**0.08**	**0.04**	**0.07**	**0.22**

## Supplementary Materials

The following are available online at http://www.mdpi.com/1424-8220/18/5/1395/s1, Figure S1: Measurement of O_2_ with Optech sensors at 1 °C. (**A**) Readings from 8 individual sensors under standard experimental conditions (1 °C, 20.86% O_2_) produced with an Optech-O_2_ Platinum reader calibrated with CalCard at 1 °C. (**B**) O_2_ readings from the same sensors measured at 1 °C (blue bar) and 22 °C (orange bar). Mean O_2_ values are 20.6 ± 0.8 and 21.0 ± 0.5, respectively. Calculated p-value of 0.12 shows that the difference is not significant (*p* > 0.05, N = 8). Temperature readings at 1 °C were 1.7 ± 1.0 °C, and at 22 °C–22.0 ± 0.1 °C.
