# Supplementary material for: Assessment of Performance of the Industrial Process of Bulk Vacuum Packaging of Raw Meat with Nondestructive Optical Oxygen Sensing Systems
Source: Sensors (Basel). 2018 May 2;18(5):1395. doi: 10.3390/s18051395 (PMC5981195; doi:10.3390/s18051395)
Supplement: Supplementary file 1 [file sensors-18-01395-s001.pdf]

Supplementary Information

A.

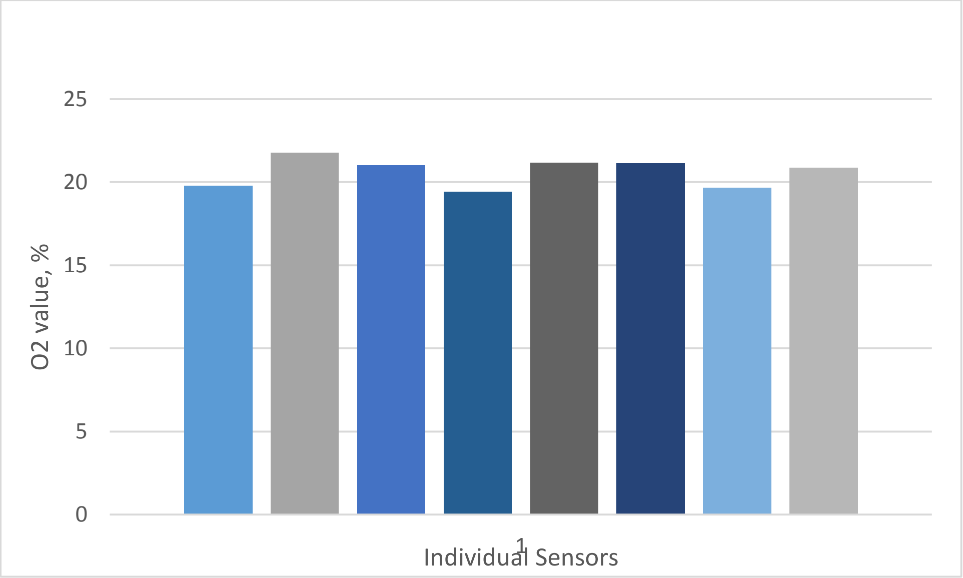

B.

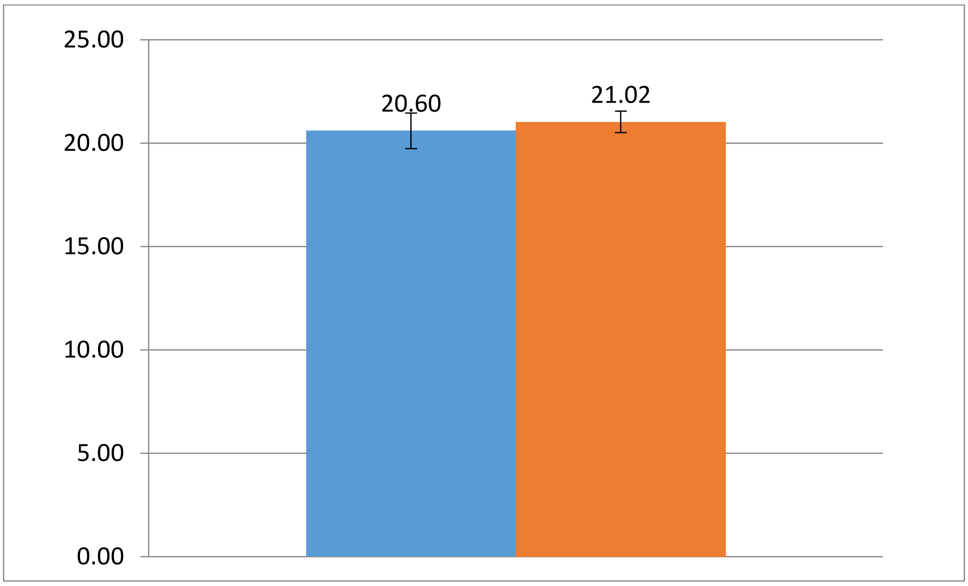

Figure S1. Measurement of O_2 with Optech sensors at 1 °C. (**A**) Readings from 8 individual sensors under standard experimental conditions (1 °C, 20.86% O_2) produced with an Optech-O_2 Platinum reader calibrated with CalCard at 1 °C. (B) O_2 readings from the same sensors measured at 1°C (blue bar) and 22 °C (orange bar). Mean O_2 values are 20.6 ± 0.8 and 21.0 ± 0.5, respectively. Calculated p-value of 0.12 shows that the difference is not significant ($p > 0.05$, N = 8). Temperature readings at 1 °C were 1.7 ± 1.0 °C, and at 22 °C–22.0 ± 0.1 °C.
